# Mapping quantitative trait loci for fruit traits and powdery mildew resistance in melon (*Cucumis melo*)

**DOI:** 10.1186/s40529-016-0130-1

**Published:** 2016-08-08

**Authors:** Yu-Hua Wang, Dong-Hong Wu, Jin-Hsing Huang, Shing-Jy Tsao, Kae-Kang Hwu, Hsiao-Feng Lo

**Affiliations:** 1 Crop Science Division, Taiwan Agricultural Research Institute, Council of Agriculture (COA), Taichung, Taiwan; 2 Plant Pathology Division, Taiwan Agricultural Research Institute, Council of Agriculture (COA), Taichung, Taiwan; 3 Department of Horticulture and Landscape Architecture, National Taiwan University, Taipei, Taiwan; 4 Department of Agronomy, National Taiwan University, Taipei, Taiwan

**Keywords:** *Cucumis melo*, Quantitative trait loci (QTL), Fruit-related traits, Fruit size, Fruit netting, *Podosphaera xanthii*

## Abstract

**Background:**

Fruit characters affect consumer preferences and the market value of melons is determined by fruit quality. Most fruit quality-related traits are controlled by multiple genes, and are influenced by environmental factors. Furthermore, powdery mildew is another limiting factor in melon production. To develop new melon cultivars with disease resistance and high quality fruits using the molecular marker-assisted breeding strategy, identification of quantitative trait loci for fruit quality and disease resistance is required.

**Results:**

The F_2_ populations from the cross of TARI-08874 (*Cucumis melo* ssp. *melo*) and ‘Bai-li-gua’ (*C*. *melo* ssp. *agrestis*) were used to map the quantitative trait loci (QTLs) for fruit-related traits and powdery mildew resistance in two trials. All traits were significantly different (P < 0.05) between parents. The generated linkage map consisted of twelve major linkage groups (LGs), spanning 626.1 cM in total, with an average distance of 8.3 cM between flanking markers. Nineteen QTLs were detected for seven melon traits, among which ten QTLs were localized to the same positions as the corresponding QTLs described in other studies. Four of these QTLs were detected in both trials. The results of identified QTLs in this study suggested that fruit size in the tested populations were mainly determined by fruit diameter and flesh thickness. All of the major QTLs for fruit diameter and flesh thickness were identified on LG5 and LG11. Four QTLs identified responsible for netting width of fruit rind were co-localized with the QTLs for netting density, suggesting similar genetic mechanisms affecting these two traits. Additionally, only one major QTL for powdery mildew resistance was detected on LG2, and it was closely linked to a simple sequence repeat (SSR) marker CMBR120 which was identified in a previous study.

**Conclusion:**

Because the netting feature is a crucial factor for external appearance of fruits in Asia market, we focus on mining the genetic information of fruit netting. This is the first report of QTL mapping to netting width. Furthermore, new QTLs were identified for netting density (*qND4*, *qND6*, and *qND7*) and netting width (*qNW2*, *qNW4*, *qNW6*, and *qNW7*) successfully. In addition, novel QTLs for fruit diameter (*qFD5*), flesh thickness (*qFT11*) were also detected.

## Background

Melon (*Cucumis melo* L.) is an economically important *Cucurbitaceae* crop widely produced in temperate and tropical regions (Fernandez-Trujillo et al. 2011). It is a diploid species with twelve chromosomes (2n = 24) (Dane 1991), and has relatively small genome size (4.5–5.0 × 10^8^ bp) (Arumuganathan and Earle 1991; Wang et al. 2006). *Cucumis melo* is classified as two subspecies, *melo* and *agrestis* (Jeffrey 1980). The former subspecies is with long hair on the hypanthium, and the later one with short hairs.

Great morphological variation exists in the fruit form, the subspecies *melo* consists of ten botanical groups (i.e., *chate, flexuosus, tibish, adana, ameri, cantalupensis, chandalak, reticulatus, inodorus,* and *dudaim*), while the subspecies *agrestis* includes five botanical groups (i.e., *acidulus, conomon, momordica, makuwa*, and *chinensis*) (Fernandez-Trujillo et al. 2011). The physiological and biochemical traits of melon fruit are also highly variable. Consumer preference and the market value of melon are affected by fruit quality in terms of shape, size, rind form, rind and flesh color, and flavor (Fernandez-Trujillo et al. 2011). For example, *cantalupensis*, *reticulatus*, and *makuwa* are consumed as a dessert fruit because of its high sugar content and excellent external characters in Taiwan. Many of these fruit quality characteristics are controlled by multiple genes and showed variable phenotypes under different environments and managements. Melon fruit size including fruit weight, fruit length, fruit diameter, and flesh thickness is polygenic control, and the QTLs for fruit size-related trait have been reported more than 67 QTLs from five populations (Eduardo et al. 2007; Monforte et al. 2004; Obando et al. 2008; Paris et al. 2008; Zalapa et al. 2007). In three populations (Obando et al. 2008; Paris et al. 2008; Ramamurthy and Waters 2015), 16 QTL were detected for fruit rind netting. It promotes marker-assisted breeding in melon. In addition, a genetic map of *C. melo* was constructed using a recombinant inbred line (RIL) population derived from *momordica* and *reticulatus* botanical groups, and fourteen QTLs for five fruit-related traits including net cover, net density, fruit length, fruit width, and fruit weight were detected (Harel-Beja et al. 2010). Recently, an F_2_ mapping population generated from crossing between the *flexuosus* and *cantalupensis* botanical groups was used to identify twelve QTLs related to four fruit traits, including fruit length, fruit width, netting density, and flesh thickness (Ramamurthy and Waters 2015).

Over the past two decades, QTL mapping have been conducted to investigate the genetic basis of melon fruit-related traits and diverse DNA-based marker systems had been applied in several different mapping populations. (Baudracco-Arnas and Pitrat 1996; Cuevas et al. 2009; Danin-Poleg et al. 2002; Fukino et al. 2012; Gonzalo et al. 2005; Oliver et al. 2001; Perin et al. 2002; Silberstein et al. 2003). These maps were constructed by different markers and different melon genotypes. It made comparative analysis among these maps difficulty, therefore, an integrated melon map combining eight linkage maps was constructed using common simple sequence repeat (SSR) markers as anchor points (Diaz et al. 2011). This consensus melon map with twelve linkage groups also integrated QTL information from eighteen mapping studies in which QTLs responsible for the resistance of various diseases, including powdery mildew, as well as fruit shape and size, rind color, fruit quality, and fruit yield (Diaz et al. 2011).

Powdery mildew is an air-borne melon disease that occurs worldwide. It is caused by *Podosphaera xanthii* (Castag.) U. Braun & N. Shish and *Golovinomyces cichoracearum* (D.C.) Huleta. Many races of powdery mildew have been identified (Jahn et al. 2002; McCreight 2006; Pitrat et al. 1998; Shishkoff 2000). In Taiwan, race 1 is the predominant *P. xanthii* (Huang and Wang 2007). Powdery mildew can occur throughout the year, particularly in enclosed space with limited ventilation. Infection of pathogens can disrupt leaf photosynthetic activity, resulting in losses in fruit yields and quality. Although the disease can be controlled by chemicals (Jahn et al. 2002; Hollomon et al. 2002), the use of resistant cultivars is favored in terms of economy and safety. To date, four genes for resistant to *P. xanthii* have been mapped, and four QTLs for powdery mildew resistance had been detected on chromosomes 2, 4, 5, and 12 (Fukino et al. 2008; Ning et al. 2014; Perchepied et al. 2005; Perin et al. 2002; Pitrat 1991; Teixeira et al. 2008; Wang et al. 2011; Yuste-Lisbona et al. 2011; Zhang et al. 2013).

The most commonly produced melon groups in Taiwan are *cantalupensis*, *reticulatus* (ssp. *melo*) and *makuwa* (ssp. *agrestis*). The production area of *makuwa* melons which have smooth and thin skin are increasing in the past several years as greenhouse cultures in the summer became more accessible and cropping systems are more flexible in Taiwan. The main factor limiting production of *makuwa* melons in Taiwan is lack of resistant variety against powdery mildew. It is considered beneficial to introduce resistant gene of powdery mildew from other melon botanical groups. Therefore, the inheritance of fruit-related traits needs to be studied in order to preserve distinct fruit morphology suited for different market classes. To our best knowledge, cross between *makuwa* melon and other densely netted melon groups has not been reported.

In this study, genetic mapping was conducted to identify QTLs attributed to phenotypic variance for powdery mildew resistance, fruit size, and fruit rind netting in melon, using an F_2_ population from the cross of a *makuwa* cultivar and an inbred line which was selected from the progenies of the cross between two cultivars, one belongs to the *cantalupensis* botanical group and the other is in the *reticulatus* group. Results from this study may provide genetic information that will contribute to breeding for cultivars with specific fruit morphology and powdery mildew resistance.

## Methods

### Plant materials and DNA extraction

**Fig. 1:**
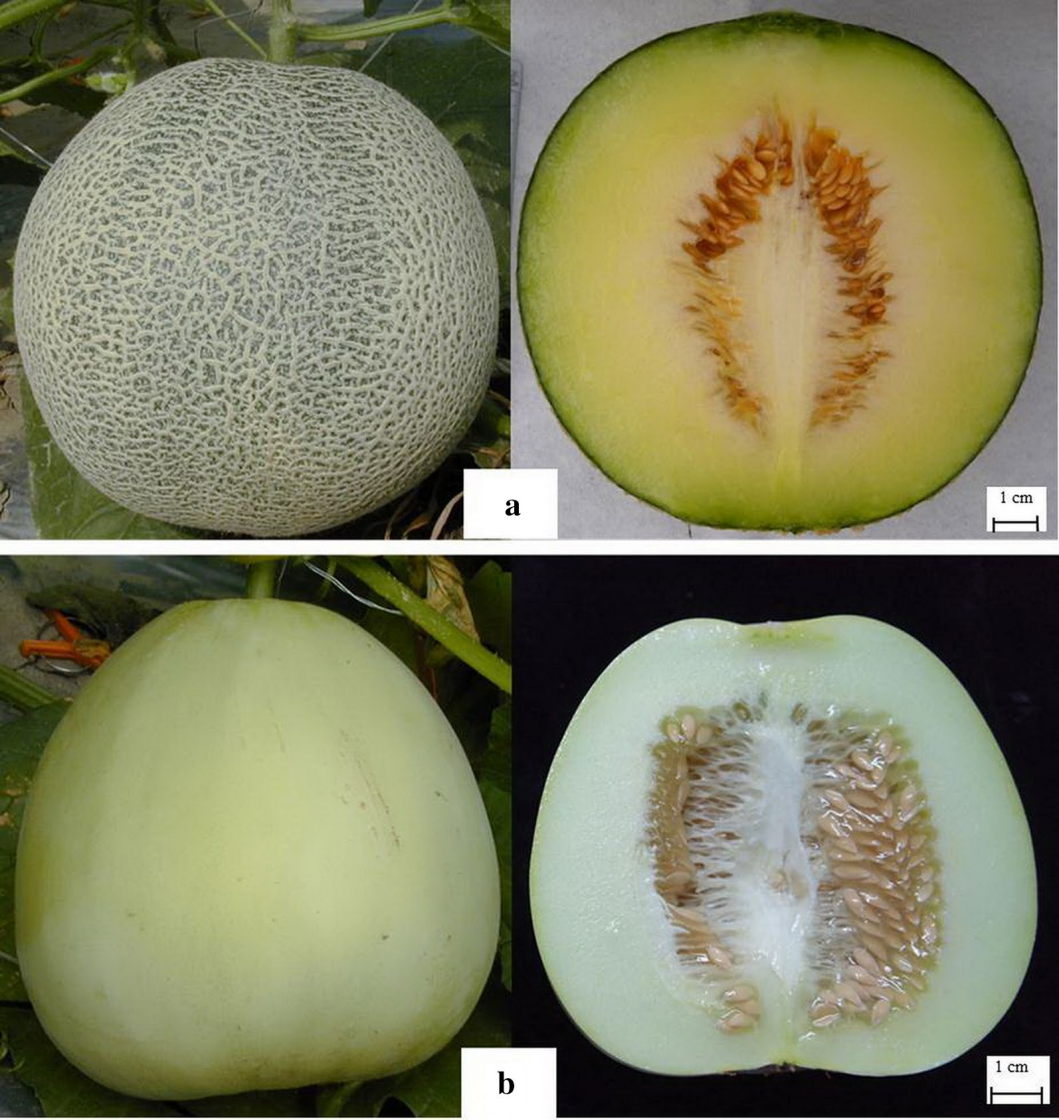
External and longitudinal sections of fruits from the parent lines. **a** TARI-08874 mature fruit. **b** ‘Bai-li-gua’ mature fruit

The F_2_ mapping population was derived from a cross between TARI-08874 and ‘Bai-li-gua’. The female parent TARI-08874 is an inbred line (i.e., self-pollinated for fifteen generations) developed from a cross between the *cantalupensis* and *reticulatus* botanical groups, with high net density, dark green rind, and green flesh (Fig. 1a), and is resistant to *P. xanthii* race 1 (Huang and Wang, 2007). The male parent ‘Bai-li-gua’ is an inbred line (i.e., self-pollinated for nine generations) that was selected from a commercial *makuwa* cultivar (ssp. *agrestis*), with a thin and smooth, white-to-light green rind (i.e., no netting) and white flesh (Fig. 1b), and is susceptible to *P. xanthii*. Two F_2_ populations, consisting of 232 and 252 plants, each originated from a selfed F_1_ plant were evaluated in two independent trials which were seeded in March and April, 2009, respectively, in a greenhouse at the Taiwan Agricultural Research Institute (TARI), Taichung, Taiwan. Twenty plants of each parent and F_1_ hybrids were evaluated along with the F_2_ populations. Total genomic DNA was extracted from young leaves from individual plant using a modified cetyltrimethylammonium bromide method (Doyle and Doyle 1990).

### Evaluation of fruit morphology and powdery mildew resistance

One fruit per plant was harvested at commercial maturity stage, which was 40–43, 29–35, and 42–43 days after anthesis for TARI-08874 (P_1_), ‘Bai-li-gua’(P_2_), and F_1_, and 26–46 days after anthesis for the F_2_ mapping populations. Six fruit-related traits were evaluated: (1) fruit weight (FW); (2) maximum fruit diameter (FD) and (3) fruit length (FL); (4) flesh thickness (FT), including the exocarp and mesocarp; (5) netting density (ND), visually scored as 0 (no netting), 1 (very slight), 2 (slight), 3 (moderate), 4 (heavy), and 5 (very heavy) (Obando et al. 2008); (6) netting width (NW), evaluated as 0 (no netting), 1 (narrow; <1 mm), 2 (moderate; 1–1.5 mm), and 3 (wide; >1.5 mm).

Powdery mildew resistance was assessed using a leaf-disc inoculation method (Cohen 1993; Huang et al. 2002). The *P. xanthii* race 1 isolate (Px-274) was provided by the Vegetable and Flower Disease Lab at the TARI. At the third leaf stage, two leaf discs (15-mm diameter) were cut from the second leaf of each plant and placed facing up in Petri dishes (90 mm) containing M-solution (10,000 ppm mannitol, 30 ppm benzimidazole, and 50 ppm tetracycline). A conidial suspension (50–100 spores/cm^2^) was uniformly sprayed over the leaf discs. The Petri dishes were then incubated at 24 °C/18 °C (day/night) with a 12-h photoperiod. After 10–14 days, the disease index (DI) was scored for each disc on a scale of 0–9, where 0 = no lesions; 1 = lesions covering 10 % of the leaf area; 3 = lesions covering 50 % of the leaf area; 5 = lesions covering 80 % of the leaf area; 7 = lesions covering 100 % of the leaf area, with thin spores on the leaf; and 9 = lesions covering 100 % of the leaf area, with a thick brown disc of sporangia on the leaf. Plants with a mean DI < 3.0 were considered resistance, while those with a mean DI ≥ 3.0 were susceptible (Epinat et al. 1993). The inoculation experiment was repeated twice at the second trial (April 2009). The means, standard deviations, results of analysis of variance for each trait, and correlations among traits were calculated using SAS Enterprise Guide 7.1 (SAS Institute Inc., Cary, North Carolina, USA).

### Simple sequence repeat marker analyses

A total of 642 *Cucumis* SSR markers were screened for polymorphisms between the parents. These included markers labeled with the following prefixes: CMACC, CMXX, CSXX (Danin-Poleg et al. 2001), CSWXX (Fazio et al. 2002), CMMS (Chiba et al. 2003), CMBR (Ritschel et al. 2004), CMXXN (Gonzalo et al. 2005), CM (Kong et al. 2007), CMN (Fukino et al. 2007), CSN (Fukino et al. 2008), ECM, GCM (Fernandez-Silva et al. 2008), ASEST25 (Al-Faifi et al. 2008), and CSJCT (Watcharawongpaiboon and Chuntongse 2008). The polymorphic SSR markers were subsequently used to genotype the F_2_ populations.

Polymerase chain reaction (PCR) was performed in a final volume of 10 μl containing 20 ng genomic DNA, 0.2 mM dNTP, 2 mM MgCl_2_, 0.2 μM SSR primers, and 0.6 U Taq DNA polymerase (Yeastern Biotech, Taiwan). The PCR was conducted in a Veriti 96-Well Thermal Cycler (Applied Biosystems, USA) using the following program: 94 °C for 2 min; two cycles of 94 °C for 1 min, 59 or 53 °C for 30 s and 72 °C for 90 s; 34 cycles of 94 °C for 20 s, 59 or 53 °C for 20 s, and 72 °C for 30 s; 72 °C for 30 min. SSR fragments of 75–320 bp were visualized on 2 % Agarose SFR (Amresco, USA) gel electrophoresis in 1× TBE buffer at 120 V for 2.5 h, and stained using SYBR Safe DNA Gel Stain (Invitrogen) for 20 min. A GeneGenius bioimaging system (Syngene, UK) was used to determine the size of SSR markers. The resulting SSR genotyping data were tested for segregation ratio of 1:2:1 in the F_2_ populations using a χ^2^ goodness-of-fit test, at a significance level of P < 0.005.

### Linkage map and quantitative trait locus analysis

The linkage map for the F_2_ population derived from TARI-08874 and ‘Bai-li-gua’ was constructed using the polymorphic SSR markers and the R statistical software (R Core Team 2013) with the qtl package (Broman et al. 2003). The SSR markers were assigned to linkage groups (LGs) based on a maximum recombination fraction of 0.34 and a minimum logarithm of odds (LOD) score of 6.0. The Kosambi map function (Kosambi 1944) was selected. The LG number was assigned according to the markers present in the consensus map (i.e., anchor markers) (Diaz et al. 2011). The ICuGI (International Cucurbit Genomics Initiative) merged map is available in the Cucurbit Genomics Database (http://www.icugi.org).

Putative QTLs controlling fruit morphological traits (i.e., FW, FL, FD, FT, ND, and NW) and powdery mildew resistance were mapped to the linkage map using R/qtl, based on the Haley–Knott regression method with 1 cM intervals, and the multiple-interval mapping method (Kao et al. 1999) for model selection. The upper 5 % quantile of 1000 permutations was used as the significance threshold. The putative QTLs were then named according to the traits and LGs.

## Results

### Fruit morphology, powdery mildew resistance, and correlation analysis

In every aspects regarding to fruit size, which including diameter, length, flesh thickness, and the overall fruit weight, TARI-08874 is significantly larger ‘Bai-li-gua’ (Table 1). The F_1_ individuals were close to or exceeding to the size of the larger parent (TARI-08874), and hence a certain level of hybrid vigor was detected.

**Table 1:** Means and standard deviations for melon traits over two trials

Trait	Trial	Parent		F_1_ plants	F_2_ population
		TARI-08874	‘Bai-li-gua’		
FW (g)	March	702.0 ± 59.7 b^a^	430.0 ± 49.9 c	786.7 ± 77.7 a	631.1 ± 197.2
	April	980.0 ± 58.9 a	600.0 ± 34.6 c	966.7 ± 100.7 b	897.3 ± 295.5
FD (cm)	March	11.1 ± 0.1 a	9.6 ± 0.3 b	10.8 ± 0.6 a	10.8 ± 1.2
	April	12.7 ± 0.4 a	10.6 ± 0.3 b	11.7 ± 0.4 a	11.5 ± 1.5
FL (cm)	March	10.2 ± 0.3 b	8.8 ± 0.9 c	12.4 ± 0.4 a	10.7 ± 1.8
	April	11.4 ± 0.4 b	10.0 ± 0.6 c	13.0 ± 0.8 a	12.6 ± 2.1
FT (cm)	March	3.1 ± 0.1 a	1.9 ± 0.2 c	2.8 ± 0.3 b	2.5 ± 0.5
	April	3.2 ± 0.5 a	2.3 ± 0.2 b	3.2 ± 0.7 a	2.7 ± 0.6
ND	March	5.0 ± 0.0 a	0 c	4.0 ± 0.0 b	2.8 ± 1.6
	April	4.0 ± 0.0 b	0 c	4.7 ± 0.6 a	2.0 ± 1.6
NW	March	3.0 ± 0.0 a	0 c	2.3 ± 0.6 b	1.4 ± 0.7
	April	3.0 ± 0.0 a	0 b	3.0 ± 0.0 a	1.3 ± 0.9
PM	March	–^b^	–	–	–
	April	0.1 ± 0.2 b	4.9 ± 0.3 a	0.4 ± 0.5 b	1.5 ± 1.8

*FW* fruit weight; *FD* fruit diameter; *FL* fruit length; *FT* flesh thickness; *ND* netting density; *NW* netting width; *PM* disease index for powdery mildew (*Podosphaera* *xanthii* race 1)

^a^Means within rows followed by different letters indicate significant differences (P < 0.05) as determined using Fisher’s protected least significant difference test

^b^Resistance to powdery mildew (*P. xanthii* race 1) was not evaluated in the trial conducted in March (i.e., Trial 1)

**Fig. 2:**
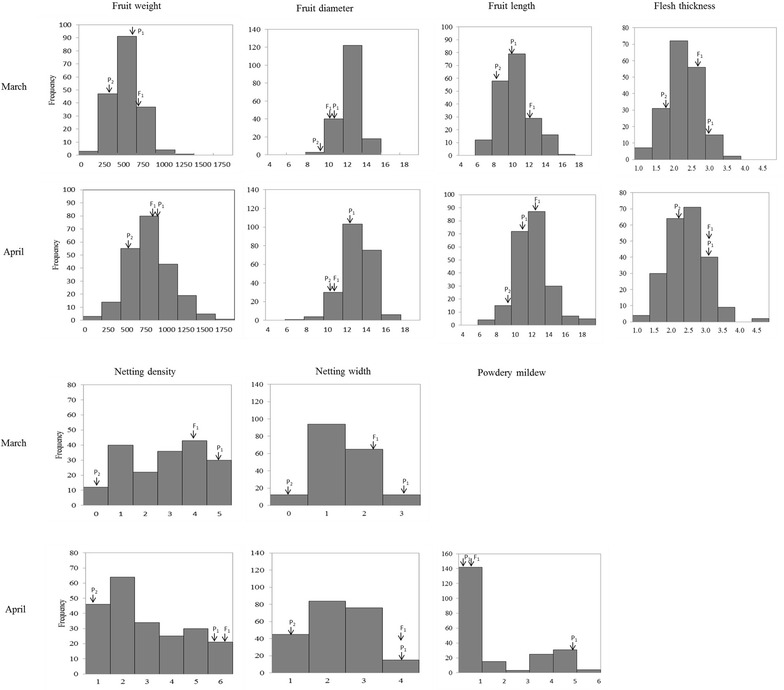
Frequency distributions of phenotypic data in the TARI-08874 × ‘Bai-li-gua’ F_2_ population over two trials. *Arrows* represent the trait value of the parental lines; TARI-08874 (P_1_), ‘Bai-li-gua’ (P_2_) and F_1_. *FW* fruit weight; *FD* fruit diameter; *FL* fruit length; *FT* flesh thickness; *ND* netting density; *NW* netting width; *PM* disease index for powdery mildew (*Podosphaera* *xanthii* race 1)

The phenotypic segregation of FW, FD, FL, and FT in the F_2_ population presented a normal distribution with some transgression, (i.e., Trial 1 in March and Trial 2 in April). While PM was skewed, the mean values of F_1_ individuals for FW in Trial 1, ND in Trial 2, and FL in both trials exhibited significant transgressions (P < 0.05). Values for FD, FT, and NW in both trials, ND in Trial 1, and powdery mildew (PM) and FW in Trial 2 were closer to those of TARI-08874 than to those of ‘Bai-li-gua’ (Table 1; Fig. 2).

Fruits were generally larger in Trial 2 than in Trial 1, as shown by the data of FW, FD, FL, and FT (Table 1). The average weight of TARI-08874 fruits was 702 and 980 g in Trials 1 and 2, respectively, while the corresponding values for ‘Bai-li-gua’ samples were 430 and 600 g in Trials 1 and 2, respectively. The TARI-08874 fruits were 10.2 and 11.4 cm long in Trials 1 and 2, respectively, while the ‘Bai-li-gua’ fruits were 8.8 cm (Trial 1) and 10 cm (Trial 2) long. The same trend was observed in the F_1_ and F_2_ populations. However, both netting characters (i.e., ND and NW) were relatively consistent between the two trials (Table 1).

The TARI-08874 parent and F_1_ population were highly resistant to *P. xanthii* race 1, whereas the ‘Bai-li-gua’ parent was susceptible. The distribution of average DI values in the F_2_ population was skewed, and the segregation of resistant and susceptible plants fit a 3:1 ratio (χ^2^ = 1.66, P > 0.05). These results suggested the resistance of TARI-08874 plants to *P. xanthii* race 1 was controlled by a single dominant gene.

To determine the relationships among the analyzed traits, a Pearson correlation coefficient analysis was performed as shown in Table 2. There were considerable correlations among the fruit size-related traits (i.e., FW, FD, FL, and FT). Results over two trials suggested FW was positively associated with FD, FL, and FT. There was also a significant association between FW, ND and NW in one trial. For fruit rind netting, ND and NW were positively correlated in both trials (r = 0.48 and 0.81 in Trials 1 and 2, respectively), suggesting that high ND was associated with relatively wide NW. Additionally, ND and NW were positively correlated with FT in both trials. The PM was negatively correlated with ND and NW because the netted TARI-08874 parent was the donor of powdery mildew resistance.

**Table 2:** Pearson correlation coefficients for fruit traits in the F_2_ population over two trials

Trait	Trial	FW	FD	FL	FT	ND	NW
FD	March	0.79**^a^					
	April	0.81**					
FL	March	0.79**	0.48**				
	April	0.76**	0.55**				
FT	March	0.70**	0.53**	0.55**			
	April	0.71**	0.64**	0.50**			
ND	March	0.09	0.01	−0.06	0.31**		
	April	0.20**	0.18**	0.04	0.40**		
NW	March	−0.04	0.00	−0.17*	0.14**	0.48**	
	April	0.22**	0.20**	0.05	0.33**	0.81**	
PM	March	–^b^	–	–	–	–	
	April	−0.05	−0.07	0.02	0.06	−0.18**	−0.18**

*FW* fruit weight; *FD* fruit diameter; *FL* fruit length; *FT* flesh thickness; *ND* netting density; *NW* netting width; *PM* disease index for powdery mildew (*Podosphaera* *xanthii* race 1)

^a^*,** Significant at P < 0.05 and P < 0.01, respectively

^b^ Resistance to powdery mildew (*P. xanthii* race 1) was not evaluated in the trial conducted in March (i.e., Trial 1)

### Genotyping using SSR and linkage map construction

**Fig. 3:**
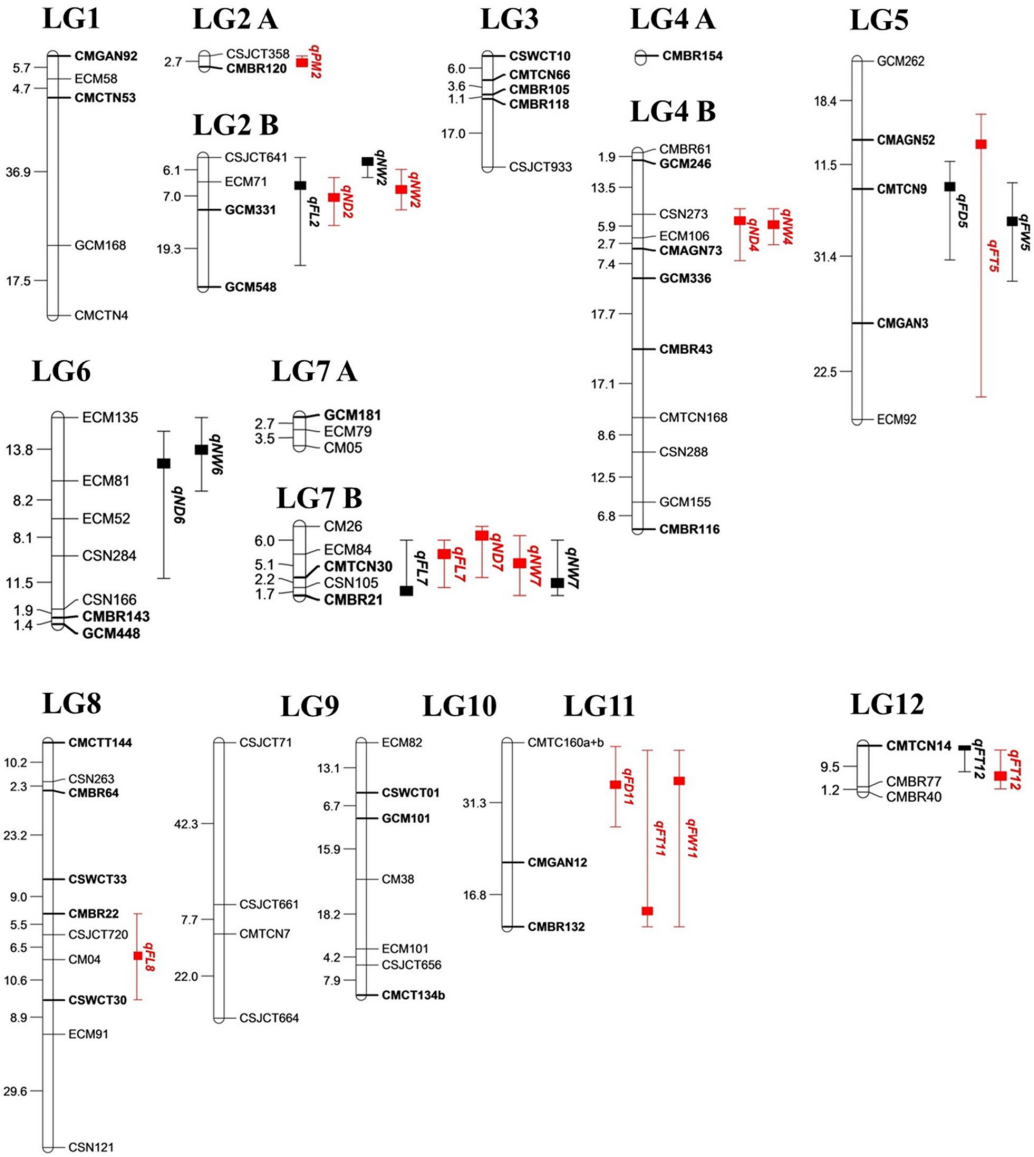
QTLs for fruit traits and disease index in the linkage map. The 95 % Bayesian confidence intervals for QTLs are indicated by extended *lines* and the positions of the highest logarithm of odds values for QTLs are indicated by *solid blocks*. *Black* and *red text* corresponds to the first and second trials, respectively. *Bold markers* are anchor markers in the consensus map (Diaz et al. 2011) or common markers on the ICuGI merged map from the Cucurbit Genomics Database (http://www.icugi.org)

Of the 642 *Cucumis* SSR markers used in this study, 102 were polymorphic between the two parents (i.e., 15.9 % polymorphism rate). The high concordance between the linkage maps constructed using two different F_2_ populations containing 232 and 252 progeny individually suggested that the recombination of traits was the similar in these two populations. Therefore, genotyping data from a total of 484 individuals combined from this two populations were applied in the linkage map construction. Determining the order of closely linked markers is prone to sampling error, and QTL mapping is sensitive to the order of markers (Broman and Sen 2009). Consequently, for closely linked markers with less than 2 cM apart, only the one with less missing data or presented in the previously published maps (anchor markers) were kept in the final linkage map (Ahfock et al. 2014). After removing some closely linked markers lacking anchor markers (i.e., genetic distances <2 cM), a linkage map consisting of 75 SSR markers was used for QTL mapping (Fig. 3). The linkage map consisted of 12 major LGs and one minor LG, spanning 626.1 cM, with an average distance of 8.3 cM between flanking markers. Two small linkage fragments containing the anchor markers CMBR120 and GCM181 and the unlinked marker CMBR154 were incorporated into LG2, LG7, and LG4, respectively, according to the marker order of the melon consensus map (Diaz et al. 2011). The minimum intervals were 1.1 cM in LG3 and 1.2 cM in LG12. Most (58 %) of the intervals between markers were smaller than 10 cM (Fig. 3). The genetic map distance ranged from 10.7 cM (LG12) to 105.8 cM (LG8), with 3 and 10 SSR markers, respectively. LG4 (including the small fragment) had 12 SSR markers, which was the most among all LGs. Except for LG9, each LG had at least one marker that was present in the consensus map of Diaz et al. (2011) for a total of 28 matching markers. Among all mapped markers, only two (i.e., 2.5 %) deviated from the Mendelian ratio (1:2:1) (P < 0.0001) in the F_2_ populations, namely GCM168 and CMCTN4 located in LG1.

### Mapping quantitative trait loci for fruit-related traits and powdery mildew resistance

Using multiple-interval mapping analysis, we detected 19 significant QTLs for seven traits over two trials (Table 3; Fig. 3). The number of QTLs ranged from one to four for each trait, and the proportion of phenotypic variance explained (PVE) by a single QTL ranged from 8.14 % (*qND6* in Trial 1) to 75.85 % (*qPM2* in Trial 2). The direction of the allelic effects at individual QTLs varied for all traits (Table 3). The QTLs were distributed in eight LGs, ranging from one in LG8 and LG12 to four in LG2 (Fig. 3). The following four QTLs (21.1 %) were detected in both trials: *qFL7* and *qNW7* (LG7), *qNW2* (LG2), and *qFT12* (LG12). In Trial 1, 10 QTLs (i.e., one each for FW, FD, FT, and ND, three for FL and NW) were detected, with LOD values ranging from 3.17 to 5.73 (Table 3). In Trial 2, 13 QTLs (i.e., one each for FW, FD, FL and PM, and three each for FT, ND, and NW) were detected, with LOD values ranging from 4.19 to 67.88. Of these QTLs, 10 (i.e., 52.6 %; *qFW5, qFW11, qFD11, qFL2, qFL7, qFL8, qFT5, qFT12, qND2,* and *qPM2*) were located at the same positions as the corresponding QTLs described in previous reports (Diaz et al. 2011, Ramamurthy and Waters 2015).

**Table 3:** Parameters of QTLs for fruit traits and powdery mildew disease index in the F_2_ population

QTL	Trait	Trial^a^	LG	Peak^b^	Flanking markers	LOD	a^c^	d	d/a	PVE %^d^	Supports previous QTLs
*qFW5*	FW	March	5	45.0	CMAGN52–CMGAN3	4.02	−104.01	−21.72	0.21	9.63	Yes
*qFW11*	FW	April	11	13.0	CMTC160a+b–CMBR132	7.04	180.65	−24.99	−0.14	13.70	Yes
*qFD5*	FD	March	5	36.3	CMAGN52–CMGAN3	3.53	−0.51	−0.02	0.05	8.50	
*qFD11*	FD	April	11	14.0	CMTC160a+b–CMGAN12	6.35	0.91	−0.08	−0.09	12.44	Yes
*qFL2*	FL	March	2	8.0	CSJCT64–GCM548	5.20	0.96	0.05	0.05	14.08	Yes
*qFL7*	FL	March	7	15.1	CM26–CMBR21	3.63	0.68	0.20	0.29	10.49	Yes
*qFL7*	FL	April	7	7.7	CM26–CMBR21	4.21	0.82	−0.24	−0.29	10.67	Yes
*qFL8*	FL	March	8	47.3	CMBR22–CSWCT30	3.83	0.53	0.74	1.40	9.94	Yes
*qFT5*	FT	April	5	14.0	GCM262–ECM92	4.19	−0.20	0.15	−0.74	9.89	Yes
*qFT11*	FT	April	11	52.0	CMTC160a+b–CMBR132	4.86	0.22	−0.16	−0.71	11.32	
*qFT12*	FT	March	12	0.0	CMTCN14–CMBR40	3.17	−0.20	0.03	−0.17	8.92	Yes
*qFT12*	FT	April	12	8.4	CMTCN14–CMBR40	4.19	−0.20	0.15	−0.83	9.89	Yes
*qND2*	ND	April	2	11.0	CSJCT641–GCM548	8.79	−0.71	0.59	−0.84	17.17	Yes
*qND4*	ND	April	4	19.0	GCM246–GCM336	8.88	0.80	−0.14	−0.18	15.01	
*qND6*	ND	March	6	12.0	ECM135–CSN166	3.38	−0.66	0.42	−0.64	8.14	
*qND7*	ND	April	7	3.0	CM26–CMTCN30	6.84	−0.61	0.63	−1.03	15.37	
*qNW2*	NW	March	2	0.0	CSJCT641–GCM331	3.76	−0.20	−0.34	1.70	11.16	
*qNW2*	NW	April	2	9.0	CSJCT641–GCM331	8.96	−0.44	0.24	−0.55	14.69	
*qNW4*	NW	April	4	19.0	GCM246–CMAGN73	11.05	0.48	−0.08	−0.17	19.55	
*qNW6*	NW	March	6	8.0	ECM135–ECM52	4.56	−0.34	−0.02	0.06	12.36	
*qNW7*	NW	March	7	14.1	CM26–CMBR21	5.73	−0.36	−0.06	0.17	14.27	
*qNW7*	NW	April	7	10.0	CM26–CMBR21	6.08	−0.30	0.27	−0.90	12.09	
*qPM2*	PM	April	2	2.7	CSJCT358–CMBR120	67.88	1.82	−1.28	−0.70	75.85	Yes

*FW* fruit weight; *FD* fruit diameter; *FL* fruit length; *FT* flesh thickness; *ND* netting density; *NW* netting width; *PM* disease index for powdery mildew (*Podosphaera* *xanthii* race 1)

^a^ Trials 1 and 2 were completed in March and April, 2009, respectively

^b^ Peak position of the significant QTL

^c^ Direction of additive effects from ‘Bai-li-gua’. d/[a] indicates degree of dominance

^d^ Phenotypic variance explained by an individual QTL

### Fruit size

Ten QTLs for fruit size-related traits were detected in LG2, LG5, LG7, LG8, LG11, and LG12 (Table 3; Fig. 3). Two of these QTLs (i.e., *qFW5* and *qFD5*) associated with FW and FD, respectively, were mapped by the same flanking markers in LG5 during Trial 1. Three other QTLs, which controlled FW, FD and FT, were mapped to LG11 in Trial 2. We identified three QTLs for FT (*qFT5, qFT11,* and *qFT12*) with a PVE value of 8.92–11.32 %. Additionally, QTLs *qFL7* and *qFT12* were detected during both trials. This study is the first to describe *qFD5* in LG5 and *qFT11* in LG11. These results suggested that FW was related with FD and FT in our population.

### Fruit rind netting

Fruit netting characters are crucial factors influencing melon quality in Asia. Netting was evaluated by two parameters in our study: netting density and netting width. The commonly used netting density (Obando et al. 2008) for fruit appearance, we devised a score measurement from 0 to 3 to describe the width of rind netting. According to the QTL analyses, *qND6* in LG6 was the only QTL mapped for ND in Trial 1. This QTL co-localized with *qNW6*, one of the four mapped QTLs for NW (Fig. 3). Three other QTLs for ND mapped during Trial 2 (i.e., *qND2*, *qND4*, and *qND7* in LG2, LG4, and LG7, respectively) co-localized with QTLs mapped for NW (i.e., *qNW2*, *qNW4*, and *qNW7*). These results further indicated a high correlation between ND and NW, with NW influencing ND. Only two QTLs (i.e., *qNW2* and *qNW7*) associated with NW were detected in both trials. New QTLs identified in this study included *qND4*, *qND6*, and *qND7* for ND and *qNW2*, *qNW4*, *qNW6*, and *qNW7* for net width.

### Powdery mildew resistance

Only one major QTL (i.e., *qPM2*) was detected for powdery mildew resistance, and it was closely linked to markers CMBR120 and CSJCT358 (Fig. 3). The LOD and PVE values were 67.88 and 75.85 %, respectively, which supported the QTL. Additionally, the CMBR120 SSR marker was located within the confidence interval of *qPM2*.

## Discussion

In our research, there were significant differences observed in fruit size-related traits between two trials, suggesting these traits were affected by growing seasons. Higher values for these traits (i.e., FW, FD, FL, and FT) were observed during Trial 2 than in Trial 1 with average temperature of 25.9 and 22.1 °C. As optimum temperature for melon growth ranging from 24 to 35 °C (Fernandez-Trujill et al. 2011), it is likely that higher temperature during the fruit development in Trial 2 was more suitable to the plant than in trial 1. However, the fruit rind netting characters (i.e., ND and NW) were relatively consistent between the two trials. In previous report (Harel-Beja et al. 2010), netting was evaluated by netting covering and netting density of melon fruit. These features were highly and positively correlated. This suggests that the fruit netting-related traits exist highly correlation.

The map constructed in this study contained twelve major linkage groups which was consistent with the consensus map reported in the previous study (Diaz et al. 2011). Besides the marker orders were consistent with those in the consensus map (Diaz et al. 2011). By the comparisons of the results of QTLs mapping with other previous reports, nine novel QTLs were identified in this study for FD, FT, ND, and NW. Nevertheless, ten out of nineteen significant QTLs detected in this study had been identified in previously studies (Diaz et al. 2011; Eduardo et al. 2007; Harel-Beja et al. 2010; Monforte et al. 2004; Obando et al. 2008; Paris et al. 2008; Ramamurthy and Waters 2015; Zalapa et al. 2007).

Because FD, FL, and FT are size factors influencing FW, the effects of *qFW5* and *qFW11* on fruit weight are likely to be the result of *qFD5, qFD11*, *qFT5*, and *qFT11*. However, we did not detect any QTLs that co-localized with QTLs for FW and FL. This suggests that fruit size in our population mainly depended on FD and FT. Similar results were reported in a previous study using a RI mapping population derived from PI414723 × Dulce (Harel-Beja et al. 2010). Two novel QTLs (i.e., *qFD5* and *qFT11*) were detected in LG5 and LG11. All fruit size-related QTLs identified in this study were reported in similar regions in previous studies of melons (Eduardo et al. 2007; Harel-Beja et al. 2010; Monforte et al. 2004; Paris et al. 2008; Zalapa et al. 2007).

Melon fruits with thick flesh are preferred by consumers (Lester 2006). However, few studies have investigated QTLs affecting FT. The mapping of QTLs for FT was first reported by Ramamurthy and Waters (2015). QTLs detected in our study co-localized with those reported by them. The QTLs for FT (i.e., *qFT5*, *qFT11*, and *qFT12*) identified in this study also detected for the trait for the thickness-to-diameter ratio reported by Obando et al. (2008) and Paris et al. (2008). There were considerable phenotypic variations for the fruit size-related traits in our F_2_ population, and the distribution of these traits suggests fruit size traits are controlled by polygenes. Our results also suggest that genomic regions in LG5 and LG11 might be important for fruit size that could be exploited in the future.

Although both trials were conducted in a greenhouse with 6 weeks apart, ambient temperatures were different because the season shifted from spring to summer in Taiwan. This change was indicated by the effective accumulated heat unit (base temperature: 15 °C) during the fruit maturation period, which went from 313 to 502 °C for TARI-08874 plants, and from 238 to 393 °C for ‘Bai-li-gua’ plants (according to meteorological data). The QTLs controlling fruit size might respond differently to temperature changes, as suggested by the QTL mapping results and significant genotype-by-trial interactions for FD and FT in a combined analysis of variance (P < 0.05) for the parental lines and F_1_ population. Similar results had been reported in previous studies (Eduardo et al. 2007; Fernandez-Trujillo et al. 2011).

Mapping of QTLs for netting width of fruit rind was unique in this study. Netting on melon surface, a wound-healing network pattern, has been reported results from a series of histological and biochemical processes (Keren-Keiserman et al. 2004; Puthmee et al. 2013). Fruit netting was scored by both netting density and netting width in which the same QTLs in LG 2, 4, 6, and 7 were identified for both traits. Because the netting characters of melon fruits serve as an important index for fruit quality in Asia market, QTLs for fruit exocarp netting had been identified (Harel-Beja et al. 2010; Paris et al. 2008; Ramamurthy and Waters 2015). In these studies, netting characters were focused on the percentage of the fruit rind covered by netting and the density of the net. Seven QTLs associated with netting coverage have been detected in LG2, LG5, LG6, LG11, and LG12 (Harel-Beja et al. 2010; Paris et al. 2008). Eight QTLs controlling ND had been identified in LG1–3 and LG8 (Harel-Beja et al. 2010; Obando et al. 2008). The QTL associated with ND in LG2 in this study (i.e., *qND2*) was previously described (Harel-Beja et al. 2010; Obando et al. 2008). However, we detected two novel QTLs associated with ND and NW, with one in LG4 and the other in LG7, from data collected in Trial 2 (i.e., warmer environment) and one QTL in LG6 from Trial 1 (i.e., cooler environment). Four NW QTLs (i.e., *qNW2*, *qNW4*, *qNW6*, and *qNW7*) were detected that co-localized with the QTLs for ND. A strong phenotypic relationship between ND and NW indicates similar genetic mechanisms regulating the two netting characters. However, the differences in the mapping results between the two trials depict significant genotype-by-trial interactions for ND and NW confirmed by the combined analysis of variance mentioned earlier. This suggests ND and NW are at least partly affected by environmental factors similar results were reported in previous studies (Keren-Keiserman et al. 2004; Puthmee et al. 2013). Two QTLs (i.e., *qNW2* and *qNW7*) were identified in both trials, indicating relatively stable ones which would be useful for MAS in the future.

Pitrat et al. (1998) identified seven *P. xanthii* races using a set of differential host melon plants. In addition, more than 28 putative *P. xanthii* races had been reported later (McCreight 2006). *P. xanthii* race 1 is the predominant race in Taiwan (Huang and Wang 2007). Our results suggest that the powdery mildew resistance exhibited by TARI-08874 is controlled by a single gene. The QTLs *qPM2* was mapped closely linked to SSR markers CMBR120 and CSJCT358. Based on the SSR markers presented in published genetic maps (Fukino et al. 2008; Ning et al. 2014; Ritschel et al. 2004; Yuste-Lisbona et al. 2011), *qPM2* was assigned to LG2. Markers CMBR120 and CMBR041 were localized to LG2 in the RIL-based map prepared by Cuevas et al. (2008), while CMBR041 was mapped to LG2 in the consensus linkage map generated by Diaz et al. (2011). These results suggest a major QTL for powdery mildew resistance in LG2 in this study. Several genes and QTLs for powdery mildew resistance had been detected on chromosomes 2, 4, 5, and 12 in previous studies (Fukino et al. 2008; Ning et al. 2014; Perchepied et al. 2005; Perin et al. 2002; Pitrat 1991; Teixeira et al. 2008; Wang et al. 2011; Yuste-Lisbona et al. 2011; Zhang et al. 2013). However, the resistance genes *Pm*-*x* (Perin et al. 2002; Pitrat 1991), *Pm*-*2F* (Zhang et al. 2013), and *Pm*-*Edisto47*-*1* (Ning et al. 2014), as well as a QTL (*PMQU2.1*) (Fukino et al. 2008), had been mapped to LG2. Based on comparison with these QTLs for powdery mildew resistance, *qPM2* is believed to be related to *PMQU2.1* from ‘AR5’ which is a powdery mildew-resistant melon cultivar (Fukino et al. 2008). The segregation ratio of the F_2_ population confirms that the powdery mildew resistance of TARI-08874 is controlled by a single dominant gene. Therefore, the F_2_ population could be used to identify the resistance gene or the relevant region in LG2. The effect of *qPM2* was confirmed in the field trials in which melon plants were inoculated with *P. xanthii* race 1. It is likely that a major gene and stable QTL mediate the powdery mildew resistance in this population. Therefore, pyramiding desirable traits to develop new high-quality melon cultivars that are resistant to powdery mildew should be possible.

## Conclusions

Our results suggest that the genetic architecture for fruit quality traits is complex and similar to other previous studies. Four QTLs for fruit length, flesh thickness, and netting width were constantly detected in two independent trials, suggesting that they are less liable to environment changes. Fruit size is associated with fruit diameter and flesh thickness, of which major QTLs were located on LG5 and LG11. In addition, high netting density is associated with wide netting width, and their QTLs are co-located on LG2, LG4, LG6, and LG7. Finally, one major QTL for powdery mildew resistance was identified in LG2 and was closely linked to the SSR marker CMBR120.

## Abbreviations

QTL: quantitative trait locus
LG: linkage group
TARI: Taiwan Agricultural Research Institute
LOD: logarithm of odds
FW: fruit weight
FD: fruit diameter
FL: fruit length
FT: flesh thickness
ND: netting density
NW: netting width
DI: disease index
PVE: phenotypic variance explained
PCR: polymerase chain reaction
